# CD4+ lymphocyte adenosine triphosphate determination in sepsis: a cohort study

**DOI:** 10.1186/cc9059

**Published:** 2010-06-11

**Authors:** Kevin L Lawrence, Patrick H White, Gerald P Morris, Jody Jennemann, Donna L Phelan, Richard S Hotchkiss, Marin H Kollef

**Affiliations:** 1 Department of Medicine, Washington University in St. Louis School of Medicine, 660 South Euclid, Campus Box 8052, St. Louis, Missouri 63110, USA; 2 Department of Pathology and Immunology, Washington University in St. Louis School of Medicine, 660 South Euclid, Campus Box 8118, St. Louis, Missouri 63110, USA; 3 HLA Laboratory, Barnes-Jewish Hospital, 1 Barnes-Jewish Hospital Plaza St. Louis, Missouri 63110, USA; 4 Department of Anesthesiology, Washington University in St. Louis School of Medicine, 660 South Euclid, Campus Box 8054, St. Louis, Missouri 63110, USA

## Abstract

**Introduction:**

Patients suffering from sepsis are currently classified on a clinical basis (i.e., sepsis, severe sepsis, septic shock); however, this clinical classification may not accurately reflect the overall immune status of an individual patient. Our objective was to describe a cohort of patients with sepsis in terms of their measured immune status.

**Methods:**

Fifty-two patients with sepsis (n = 13), severe sepsis (n = 21), or septic shock (n = 18) were studied. The immune status was determined by measuring the CD4+ lymphocyte adenosine triphosphate (ATP) content after mitogen stimulation in whole blood.

**Results:**

The measured CD4+ lymphocyte ATP content at the time of ICU admission did not differ among the various groups defined by the sepsis classification system (sepsis = 454 ± 79 ng/ml; severe sepsis = 359 ± 54 ng/ml; septic shock = 371 ± 53 ng/ml; *P *= 0.44). Furthermore, survivors of sepsis had a significantly higher CD4+ lymphocyte ATP content at the time of ICU admission than did nonsurvivors of sepsis (431 ± 41 ng/mL vs. 266 ± 53 ng/mL, respectively; *P *= 0.04).

**Conclusions:**

The sepsis classification system that is currently used is not representative of the individual immune status as determined by measuring the CD4+ lymphocyte ATP content. Moreover, a lower CD4+ ATP content at the time of ICU admission is associated with a worse clinical outcome in those suffering from sepsis.

## Introduction

Sepsis, the systemic inflammatory response syndrome that results from infection, is associated with considerable mortality. Besides controlling the inciting infectious insult and providing good supportive care, few therapies are available to treat this syndrome. Although it seems conceptually appealing that suppressing the generalized inflammatory response in sepsis would improve outcomes, the evaluation of numerous adjuvant therapies has led to conflicting, yet disappointing results [1-3].

Currently, patients suffering from sepsis are classified based on the presence of organ dysfunction and/or shock (i.e., sepsis, severe sepsis, septic shock). Although this classification system is widely used, it may not accurately reflect the overall immune status of an individual patient (i.e., hypoimmune, hyperimmune) [4]. This is especially important because the current sepsis treatment guidelines recommend consideration of the prescription of immune altering therapy (i.e., corticosteroids) based on the clinical classification of septic shock [5]. Furthermore, corticosteroids alter cell-mediated immune function, and evidence suggests that depressed cell-mediated immune function is associated with worse clinical outcomes [6-9]; therefore, a biological marker of cell-mediated immune status could have the potential to improve classification and risk stratification in sepsis.

We hypothesized that the evaluation of a biomarker used to determine the cell-mediated immune status would be informative in a cohort of patients with sepsis. Furthermore, we hypothesized that their measured immune status might not be reflective of their sepsis classification. Finally, we hypothesized that their immune status might be associated with mortality.

## Materials and methods

### Patient population and study setting

This study was approved by the Institutional Review Board at Washington University School of Medicine Human Studies Committee. Patients were enrolled in the study within 24 hours of admission to the medical ICUs at Barnes-Jewish Hospital in St. Louis, Missouri. In addition, patients met the criteria for sepsis (n = 13), severe sepsis (n = 21), or septic shock (n = 18) as defined according to the American College of Chest Physicians/Society of Critical Care Medicine Consensus Conference [10]. All patients, or an acceptable surrogate, provided informed consent to participate in the study.

Patients deemed likely to have a poor outcome, for a reason known at admission other than sepsis, severe sepsis, or septic shock (i.e., cardiac arrest or widely metastatic cancer) were not enrolled in the study. Patients were followed until hospital discharge, and their routine clinical and laboratory data were recorded. All patients received early goal-directed therapy according to a standard protocol emphasizing adequate volume administration, appropriate antibiotic administration, and optimal oxygen delivery.

### Specimen collection

**Figure 1:**
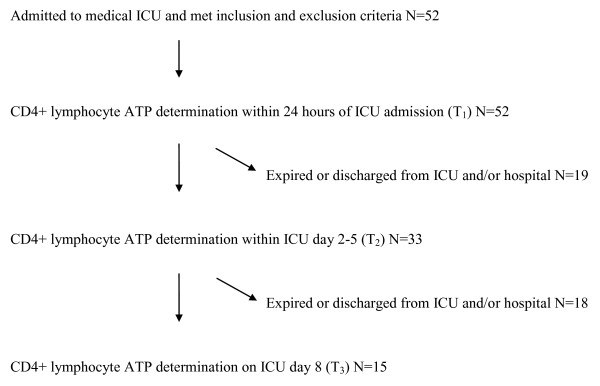
**Time frame of serial CD4+ lymphocyte ATP content and patient flow during investigation**.

Blood was collected into sodium heparin tubes (Vacutainer; Becton Dickinson, Franklin Lakes, NJ, USA) through existing venous catheters in all patients. This collection was performed at serial time points during the course of each patient's ICU stay. Specifically, blood was collected at T_1 _= ICU day 1 (admission), T_2 _= ICU day 2 to 5, and T_3 _= ICU day 8 (Figure 1). After collection, the blood was then transported, at room temperature, to the laboratory for same day processing. Blood specimens were collected only during the course of the ICU stay.

### CD4+ lymphocyte ATP content determination

The Immuknow^® ^assay (Cylex Inc., Columbia, MD, USA) was used to determine the ATP content of CD4+ lymphocytes and was performed according to the manufacturer's instructions and previous descriptions [11,12]. Briefly, 100 μL of a 1:4 dilution of whole blood was incubated with phytohemagglutinin (2.5 μg/mL) for 15 to 18 hours in a 5% carbon dioxide incubator at 37°C. Anti-human CD4 monoclonal antibody-coated magnetic particles (Dynal, Oslo, Norway) were added to select CD4+ cells after stimulation. After washing the selected CD4+ cells selected on a strong magnet (Cylex Cat. 1050; Cylex Inc., Columbia, MD, USA), a lysing reagent was added to release intracellular ATP. A luciferin/luciferase mixture was then added to the cell lysate. Within 30 minutes after the addition of enzyme, the bioluminescent product was measured in a luminometer (PHL Mediators, Austria, or Berthold, Maryville, TN, USA, or Turner Designs, Sunnyvale, CA, USA). The amount of light emitted (emission maximum 562 nm) was compared with a calibration curve generated with ATP calibrators (0, 1, 10, 100, and 1,000 ng/mL). The concentration of ATP (ng/mL) in each sample was then calculated from the calibration curve using an Excel-based program provided by Cylex (Columbia, MD, USA). Replicate samples with a calculated percentage coefficient of variation greater than 20% were included in the calculation if a single value was within three standard deviations of the mean value of all wells.

### Statistical analysis

All data are presented as the mean ± the standard error of the mean unless otherwise indicated. All comparisons were unpaired and all tests of significance were two-tailed. SPSS version 11.0 for Windows (SPSS, Inc., Chicago, IL, USA) was used for statistical analysis. Continuous variables were compared using the Mann-Whitney U test or the Kruskal-Wallis test, where appropriate, for non-normally distributed variables. The Pearson chi-squared was used to compare categorical variables. A *P*-value of less than 0.05 was considered significant.

### Role of the sponsor

The study sponsor, Cylex, Inc., had no role in the design of the study. They also had no role in the collection or interpretation of data, and they had no role in the preparation of the manuscript or the decision to submit it for publication.

## Results

### Characteristics of the study population

We evaluated 52 patients in our investigation. Overall, these patients appear representative of typical patients with sepsis encountered in an ICU setting. Table 1 shows the main characteristics of our study population. Thirteen patients were considered to be immunocompromised based on one or more of the following criteria: HIV positivity, organ transplant recipient, pharmacologic immunosuppression, recent chemotherapy (within eight weeks prior to enrollment).

**Table 1:** Characteristics of study population (n = 52)

Characteristic	Value
Age (years)	57 ± 2
Sex, n (%)	
Male	27 (52)
Female	25 (48)
Race, n (%)	
White	32 (62)
African American	20 (38)
Admitting sepsis classification, n (%)	
Sepsis	13 (25)
Severe sepsis	21 (40)
Septic shock	18 (35)
Source of sepsis	
Lung	27 (52)
Abdomen (including urinary source)	15 (29)
Blood	5 (10)
Other	5 (10)
Acute Physiology and Chronic Health Evaluation II Score	22 ± 1
Source of admission	
Emergency department	26 (50)
Hospital ward	18 (35)
Other	8 (15)
Length of stay (days)	
ICU	5 ± 1
Hospital	13 ± 2
Culture results, n (%)	
Positive	34 (65)
Negative	18 (35)
Immune status, n (%)	
Immunocompetent	39 (75)
Immunocompromised	13 (25)

### Clinical and laboratory data according to conventional sepsis classification

**Figure 2:**
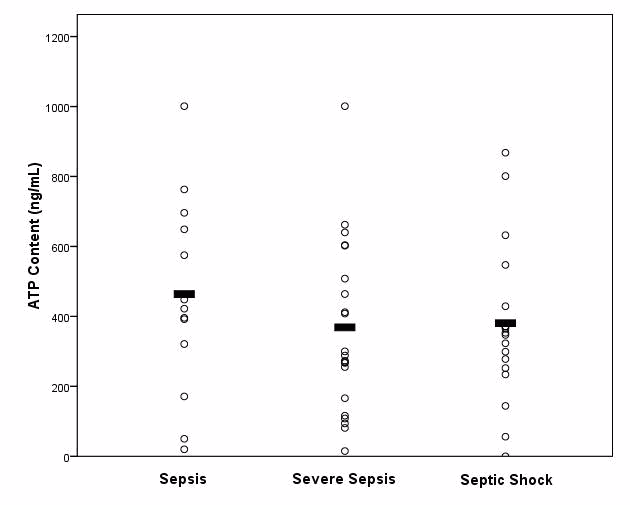
**CD4+ lymphocyte ATP content at ICU admission among patients when grouped according to the sepsis classification system**. *P *= 0.44. Black bars indicate the mean CD4+ lymphocyte ATP content for each group, respectively.

Figure 2 demonstrates the CD4+ lymphocyte ATP content according to the conventional sepsis classification at the time of ICU admission. There was no significant difference in the CD4+ lymphocyte ATP content among patients when grouped according to this classification system (sepsis = 454 ± 79 ng/ml; severe sepsis = 359 ± 54 ng/ml; septic shock = 371 ± 53 ng/ml; *P *= 0.44). In addition, there was no significant difference in the total white blood cell count among the different groups (sepsis = 16.4 ± 3.4 × 10^3^cells/μL; 16.8 ± 2.0 × 10^3^cells/μL; 16.9 ± 2.5 × 10^3^cells/μL; *P *= 0.82), and the absolute lymphocyte count was also not significantly different among the groups (1.9 ± 0.9 × 10^3 ^cells/μL; 1.0 ± 0.1 × 10^3 ^cells/μL; 2.3 ± 1.4 × 10^3 ^cells/μL, respectively; *P *= 0.98). Not surprisingly, the Acute Physiology and Chronic Health Evaluation (APACHE) II score did differ among the groups (16.4 ± 1.6; 23.7 ± 1.4; 25.1 ± 2.1, respectively; *P *= 0.006); however, the mortality difference among the groups did not reach statistical significance (23.1%; 23.8%; 33.3%, respectively; *P *= 0.75).

### Clinical and laboratory data according to clinical immune status

The mean CD4+ lymphocyte ATP content differed significantly between the immunocompetent group of patients and the immunocompromised group of patients (437 ± 38 ng/mL vs. 237 ± 63 ng/mL, respectively; *P *= 0.01). However, the total white blood cell count was not significantly different between these groups(16.8 ± 1.4 × 10^3^cells/μL vs. 16.6 ± 3.9 × 10^3^cells/μL, respectively; *P *= 0.65), and the absolute lymphocyte count was also not significantly different (1.3 ± 0.3 × 10^3 ^cells/μL vs. 2.7 ± 1.9 × 10^3 ^cells/μL, respectively; *P *= 0.27). Furthermore, the APACHE II score did not differ between the groups (22.2 ± 1.3 vs. 22.9 ± 2.3, respectively; *P *= 1.00), and the mortality also did not differ significantly (25.6% vs. 30.8%, respectively; *P *= 0.73).

### Clinical and laboratory data according to mortality

Table 2 demonstrates the mortality stratified by quartiles of measured CD4+ lymphocyte ATP content. Clearly there are differences in the mortality when patients are stratified in this manner; however, these differences did not reach statistical significance (*P *= 0.21). The total white blood cell count was also not significantly different between survivors and nonsurvivors (16.4 ± 1.4 vs. 17.5 ± 3.7 × 10^3^cells/μL, respectively; *P *= 0.87). In addition, the absolute lymphocyte count was not significantly different between the survivors and nonsurvivors (2.0 ± 0.7 vs. 0.8 ± 0.2 × 10^3 ^cells/μL, respectively; *P *= 0.08). Not surprisingly, the APACHE II score did differ between survivors and nonsurvivors (19.8 ± 1.0 vs. 29.4 ± 2.3, respectively; *P *< 0.0001).

**Table 2:** Mortality by quartile of CD4+ lymphocyte ATP content measure at the time of ICU admission

ATP content (ng/mL)	Nonsurvivors/Total	Mortality (%)
≤250	6/13	46
251-500	6/24	25
501-750	2/10	20
≥751	0/5	0

Statistical significance for mortality was *P *> 0.05 for comparisons between quartiles.

**Figure 3:**
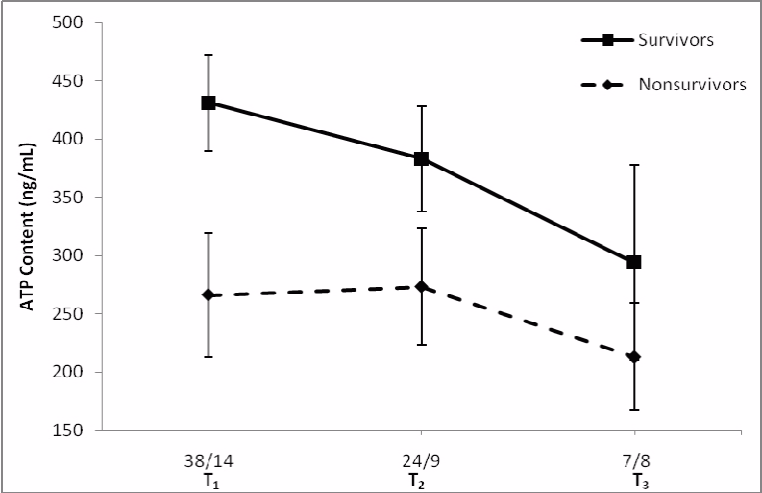
**Serial measurements of CD4+ lymphocyte ATP content during the course of ICU stay according to survival status**. Data are shown for T_1 _= ICU day 1, T_2 _= ICU day 2 to 5, and T_3 _= ICU day 8. Numbers presented under the horizontal axis represent the number of patients in the survivor and nonsurvivor groups at the different time points, respectively. * *P *< 0.05 for T_1_; *P *> 0.05 for T_2 _and T_3_.

Figure 3 demonstrates the serial measurements of CD4+ lymphocyte ATP content over time during the ICU stay grouped according to survivor and nonsurvivor status. There was a significant difference in CD4+ lymphocyte ATP content at admission, T_1_, between survivors and nonsurvivors (431 ± 41 ng/mL vs. 266 ± 53 ng/mL, respectively; *P *= 0.04). Although differences were noted in CD4+ lymphocyte ATP content at other time points, statistical significance was not demonstrated other than at admission (T_1_); however, fewer patients were evaluated at subsequent time points.

## Discussion

We have demonstrated that there is a wide range of CD4+ lymphocyte ATP content in a typical ICU population suffering from sepsis. Furthermore, we have demonstrated that the current sepsis classification (i.e., sepsis; severe sepsis; septic shock) does not accurately reflect the CD4+ lymphocyte ATP content, and arguably, the immune status of an individual patient. We have also demonstrated that a lower CD4+ lymphocyte ATP content is associated with a higher mortality, and most surprisingly, this finding is present at the time of ICU admission.

Measuring CD4+ lymphocyte ATP content after exposure to a stimulus is one method of determining the global cell-mediated immune response, and this approach has primarily been used as an aid to guide immunosuppressive therapy in transplant recipients [12,13]. In theory, a biomarker such as this could yield valuable information in staging the host immune response in the setting of sepsis, and it could potentially identify those who could benefit, or be harmed, by a particular intervention [14]. For example, one might surmise that administering a therapy that lowers the immune status (i.e, corticosteroids) based solely on the presence of shock -- when in fact the immune status may already be markedly suppressed -- could have an undesired effect because of improper patient selection.

Our data are consistent with previously published results suggesting that decreased cell-mediated immune function is associated with a worse prognosis in the setting of sepsis. For example, Heidecke and colleagues demonstrated that decreased T cell proliferation correlated with mortality in patients with post-operative sepsis due to intraabdominal infections [8]. Furthermore, Meakins and colleagues used reactivity to skin testing to evaluate the host immune response and found decreased reactivity to be associated with mortality in several patient populations [9].

Interestingly, data are beginning to accumulate supporting the notion that viruses may reactivate in immunocompetent patients during times of critical illness, and this reactivation seems to be associated with worse clinical outcomes, including mortality [6,7]. In fact, it is conceivable that these pathogens may represent an epiphenomenon of an underlying 'acquired immunosuppressed state'; however, this is not to say that these pathogens may not be capable of causing end-organ disease (i.e., true infections). To further support this notion, the mean CD4+ lymphocyte ATP content from our sepsis nonsurvivors is similar to the reported mean CD4+ lymphocyte ATP content of solid-organ transplant recipients (266 vs. 282, respectively) [12]. Similarly, the mean CD4+ lymphocyte ATP content from our sepsis survivors is similar to the reported mean CD4+ lymphocyte ATP content of healthy controls (431 vs. 432, respectively) [12].

The reason for why lower CD4+ lymphocyte ATP content is associated with a worse prognosis is strictly speculation, yet interesting. First, this decrease may be due to mitochondrial dysfunction [15-19]. Second, this decrease may be due to anergy of the lymphocytes to mitogen stimulation [8,20]. It should also be noted that this decrease may simply represent another form of organ dysfunction -- lymphocyte bioenergetic failure -- in the setting of sepsis.

Our study has several limitations that should be mentioned. First, the CD4+ lymphocyte counts were not determined, so the measured CD4+ lymphocyte ATP content could be an indirect measure of cell number. However, previous experience using this assay in solid-organ transplant recipients indicates that this is unlikely to be the case, and we have reported the total lymphocyte counts, which also support this notion. Also, our small sample size limits our ability to draw definitive conclusions regarding the use of this assay in staging the host response to sepsis.

On the other hand, our study also has several strengths. To our knowledge, this is the first investigation to use this method in an attempt to stage the host immune response in sepsis; therefore, our findings are important when viewed as thought provoking and hypothesis generating. Furthermore, patients in our study classified as immunocompromised based on clinical criteria had significantly different assay results than those who were immunocompetent, which is similar to prior published data [12]. This observed difference further supports the notion that this assay is an objective marker of the global cell-mediated immune status of an individual, and therefore, provides useful information, particularly in the setting of sepsis.

In summary, we have demonstrated that the sepsis classification system that is currently used does not accurately reflect the immune status of an individual when measured by determining the CD4+ lymphocyte ATP content. This finding raises the question, 'Is the current sepsis classification system reliable at determining the immune status, specifically cell-mediated immune status, and should this classification system be used to direct adjuvant immunomodulatory therapy in the setting of sepsis?'. Furthermore, we have demonstrated that a lower CD4+ ATP content is associated with a worse clinical outcome in those suffering from sepsis, and importantly, this finding is present at the time of ICU admission.

## Conclusions

The sepsis classification system that is currently used is not representative of the individual immune status as determined by measuring the CD4+ lymphocyte ATP content. Moreover, a lower CD4+ ATP content at the time of ICU admission is associated with a worse clinical outcome in those suffering from sepsis.

## Key messages

• Sepsis is the systemic inflammatory response that results from an infectious insult.

• Patients with sepsis are currently classified based on the presence of organ dysfunction and/or shock (i.e., sepsis, severe sepsis, septic shock).

• The immune status of an individual patient may not correlate with the current classification system that is used to categorize these patients.

• Our data shows no significant difference in the measured immune status of patients based on the current classification system.

• Patients with sepsis who have lower measures of immune function at the time of ICU admission appear to have an increased mortality.

## Abbreviations

APACHE II: acute physiology and chronic health II.

## Competing interests

This study was supported in part by Cylex Inc. through donations of laboratory equipment and supplies. The study sponsor, Cylex, Inc., had no role in the design of the study. They also had no role in the collection or interpretation of data, and they had no role in the preparation of the manuscript or the decision to submit it for publication.

## Authors' contributions

KLL, PHW, GPM, JJ, DLP, RSH, and MHK all contributed to the conception and design of the study or the acquisition of data or analysis and interpretation of the data. All were involved in drafting the manuscript or revising it for intellectual content. All gave final approval to the version of the manuscript to be published.

## References

[B1] Bone RC Why sepsis trials fail JAMA 1996 276 565 566 10.1001/jama.276.7.565 8709407

[B2] Freeman BD Natanson C Anti-inflammatory therapies in sepsis and septic shock Expert Opin Investig Drugs 2000 9 1651 1663 10.1517/13543784.9.7.1651 11060768

[B3] Sprung CL Annane D Keh D Moreno R Singer M Freivogel K Weiss YG Benbenishty J Kalenka A Forst H Laterre PF Reinhart K Cuthbertson BH Payen D Briegel J CORTICUS Study Group Hydrocortisone therapy for patients with septic shock N Engl J Med 2008 358 111 124 10.1056/NEJMoa071366 18184957

[B4] Bone RC Grodzin CJ Balk RA Sepsis: a new hypothesis for pathogenesis of the disease process Chest 1997 112 235 243 10.1378/chest.112.1.235 9228382

[B5] Dellinger RP Levy MM Carlet JM Bion J Parker MM Jaeschke R Reinhart K Angus DC Brun-Buisson C Beale R Calandra T Dhainaut JF Gerlach H Harvey M Marini JJ Marshall J Ranieri M Ramsay G Sevransky J Thompson BT Townsend S Vender JS Zimmerman JL Vincent JL International Surviving Sepsis Campaign Guidelines Committee, American Association of Critical-Care Nurses, American College of Chest Physicians, American College of Emergency Physicians, Canadian Critical Care Society, European Society of Clinical Microbiology and Infectious Diseases, European Society of Intensive Care Medicine, European Respiratory Society, International Sepsis Forum, Japanese Association for Acute Medicine, Japanese Society of Intensive Care Medicine, Society of Critical Care Medicine, Society of Hospital Medicine, Surgical Infection Society, World Federation of Societies of Intensive and Critical Care Medicine Surviving Sepsis Campaign: international guidelines for management of severe sepsis and septic shock: 2008 Crit Care Med 2008 36 296 327 10.1097/01.CCM.0000298158.12101.41 18158437

[B6] Limaye AP Kirby KA Rubenfeld GD Leisenring WM Bulger EM Neff MJ Gibran NS Huang ML Santo Hayes TK Corey L Boeckh M Cytomegalovirus reactivation in critically ill immunocompetent patients JAMA 2008 300 413 422 10.1001/jama.300.4.413 18647984 PMC2774501

[B7] Luyt CE Combes A Deback C Aubriot-Lorton MH Nieszkowska A Trouillet JL Capron F Agut H Gibert C Chastre J Herpes simplex virus lung infection in patients undergoing prolonged mechanical ventilation Am J Respir Crit Care Med 2007 175 935 942 10.1164/rccm.200609-1322OC 17234903

[B8] Heidecke CD Hensler T Weighardt H Zantl N Wagner H Siewert JR Holzmann B Selective defects of T lymphocyte function in patients with lethal intraabdominal infection Am J Surg 1999 178 288 292 10.1016/S0002-9610(99)00183-X 10587185

[B9] Meakins JL Pietsch JB Bubenick O Kelly R Rode H Gordon J MacLean LD Delayed hypersensitivity: indicator of acquired failure of host defenses in sepsis and trauma Ann Surg 1977 186 241 250 10.1097/00000658-197709000-00002 142452 PMC1396336

[B10] Anonymous American College of Chest Physicians/Society of Critical Care Medicine Consensus Conference: definitions for sepsis and organ failure and guidelines for the use of innovative therapies in sepsis Crit Care Med 1992 20 864 874 10.1097/00003246-199206000-00025 1597042

[B11] ImmuKnow^®^: the Cylex^® ^Immune Cell Function Assay http://www.cylex.net/pdf/ImmuKnow_Insert-cx.pdf

[B12] Kowalski R Post D Schneider MC Britz J Thomas J Deierhoi M Lobashevsky A Redfield R Schweitzer E Heredia A Reardon E Davis C Bentlejewski C Fung J Shapiro R Zeevi A Immune cell function testing: an adjunct to therapeutic drug monitoring in transplant patient management Clin Transplant 2003 17 77 88 10.1034/j.1399-0012.2003.00013.x 12709071

[B13] Kowalski RJ Post DR Mannon RB Sebastian A Wright HI Sigle G Burdick J Elmagd KA Zeevi A Lopez-Cepero M Daller JA Gritsch HA Reed EF Jonsson J Hawkins D Britz JA Assessing relative risks of infection and rejection: a meta-analysis using an immune function assay Transplantation 2006 82 663 668 10.1097/01.tp.0000234837.02126.70 16969290

[B14] Marshall JC Reinhart K International Sepsis Forum Biomarkers of sepsis Crit Care Med 2009 37 2290 2298 10.1097/CCM.0b013e3181a02afc 19487943

[B15] Adrie C Bachelet M Vayssier-Taussat M Russo-Marie F Bouchaert I Adib-Conquy M Cavaillon JM Pinsky MR Dhainaut JF Polla BS Mitochondrial membrane potential and apoptosis peripheral blood monocytes in severe human sepsis Am J Respir Crit Care Med 2001 164 389 395 11500338 10.1164/ajrccm.164.3.2009088

[B16] Brealey D Brand M Hargreaves I Heales S Land J Smolenski R Davies NA Cooper CE Singer M Association between mitochondrial dysfunction and severity and outcome of septic shock Lancet 2002 360 219 223 10.1016/S0140-6736(02)09459-X 12133657

[B17] Crouser ED Mitochondrial dysfunction in septic shock and multiple organ dysfunction syndrome Mitochondrion 2004 4 729 741 10.1016/j.mito.2004.07.023 16120428

[B18] Fredriksson K Hammarqvist F Strigard K Hultenby K Ljungqvist O Wernerman J Rooyackers O Derangements in mitochondrial metabolism in intercostal and leg muscle of critically ill patients with sepsis-induced multiple organ failure Am J Physiol Endocrinol Metab 2006 291 E1044 1050 10.1152/ajpendo.00218.2006 16803854

[B19] Carre JE Singer M Cellular energetic metabolism in sepsis: the need for a systems approach Biochim Biophys Acta 2008 1777 763 771 10.1016/j.bbabio.2008.04.024 18482575

[B20] Hotchkiss RS Karl IE The pathophysiology and treatment of sepsis N Engl J Med 2003 348 138 150 10.1056/NEJMra021333 12519925

